# Prognostic Utility of Biomarkers in Predicting of One-Year Outcomes in Patients with Aortic Stenosis Treated with Transcatheter or Surgical Aortic Valve Implantation

**DOI:** 10.1371/journal.pone.0048851

**Published:** 2012-12-14

**Authors:** Jiri Parenica, Petr Nemec, Josef Tomandl, Jiri Ondrasek, Monika Pavkova-Goldbergova, Martin Tretina, Jiri Jarkovsky, Simona Littnerova, Martin Poloczek, Petr Pokorny, Jindrich Spinar, Zdenka Cermakova, Roman Miklik, Petr Malik, Ondrej Pes, Jolana Lipkova, Marie Tomandlova, Petr Kala

**Affiliations:** 1 University Hospital Brno, Brno, Czech Republic; 2 Medical Faculty, Masaryk University, Brno, Czech Republic; 3 International Clinical Research Center–Department of Cardiovascular Disease, University Hospital St. Anne's, Brno, Czech Republic; 4 Center of Cardiovascular Surgery and Transplantations, Brno, Czech Republic; 5 Institute of Biochemistry, Medical Faculty, Masaryk University, Brno, Czech Republic; 6 Institute of Pathological Physiology, Faculty of Medicine, Masaryk University, Brno, Czech Republic; 7 Institute of Biostatistics and Analyses, Faculty of Medicine, Masaryk University, Brno, Czech Republic; 8 Biochemistry Department, Faculty Hospital Brno, Brno, Czech Republic; 9 Institute of Laboratory Methods, Masaryk University, Brno, Czech Republic; University of Sao Paulo Medical School, Brazil

## Abstract

**Objectives:**

The aim of the work was to find biomarkers identifying patients at high risk of adverse clinical outcomes after TAVI and SAVR in addition to currently used predictive model (EuroSCORE).

**Background:**

There is limited data about the role of biomarkers in predicting prognosis, especially when TAVI is available.

**Methods:**

The multi-biomarker sub-study included 42 consecutive high-risk patients (average age 82.0 years; logistic EuroSCORE 21.0%) allocated to TAVI transfemoral and transapical using the Edwards-Sapien valve (n = 29), or SAVR with the Edwards Perimount bioprosthesis (n = 13). Standardized endpoints were prospectively followed during the 12-month follow-up.

**Results:**

The clinical outcomes after both TAVI and SAVR were comparable. Malondialdehyde served as the best predictor of a combined endpoint at 1 year with AUC (ROC analysis) = 0.872 for TAVI group, resp. 0.765 (p<0.05) for both TAVI and SAVR groups. Increased levels of MDA, matrix metalloproteinase 2, tissue inhibitor of metalloproteinase (TIMP1), ferritin-reducing ability of plasma, homocysteine, cysteine and 8-hydroxy-2-deoxyguanosine were all predictors of the occurrence of combined safety endpoints at 30 days (AUC 0.750–0.948; p<0.05 for all). The addition of MDA to a currently used clinical model (EuroSCORE) significantly improved prediction of a combined safety endpoint at 30 days and a combined endpoint (0–365 days) by the net reclassification improvement (NRI) and the integrated discrimination improvement (IDI) (p<0.05).

Cystatin C, glutathione, cysteinylglycine, asymmetric dimethylarginine, nitrite/nitrate and MMP9 did not prove to be significant. Total of 14.3% died during 1-year follow-up.

**Conclusion:**

We identified malondialdehyde, a marker of oxidative stress, as the most promising predictor of adverse outcomes during the 30-day and 1-year follow-up in high-risk patients with symptomatic, severe aortic stenosis treated with TAVI. The development of a clinical “TAVIscore” would be highly appreciated. Such dedicated scoring system would enable further testing of adjunctive value of various biomarkers.

## Introduction

Transcatheter aortic valve implantation (TAVI) is a treatment option for patients with severe aortic stenosis (AS), who are considered poor candidates for surgical aortic valve replacement (SAVR) primarily due to comorbidities. According to a previous study, 30-day and 6-month survival rates following TAVI and SAVR were comparable, but TAVI was associated with shorter operation, intubation and intensive care unit times [1]. The selection of patients was based on expected perioperative or short-term mortality that is usually calculated from the two most common risk models – the logistic EuroSCORE [2] or the Society of Thoracic Surgeons Predicted Risk of Mortality (STS-PROM) algorithms. Using the logistic EuroSCORE markedly overestimated the risk of very high-risk patients [3], [4] and in 2011 a new logistic EuroSCORE II was presented. At this point we must underline the fact that these scoring systems were designed to predict only 30-day mortality rates for patients undergoing cardiac surgery. At present, no dedicated scoring system is available for patients treated with TAVI. The one-year mortality rate of conservatively treated high-risk patients with severe AS is 50%. Despite either SAVR or TAVI treatment, the one-year mortality rate still remains relatively high - 20–30% [5]–[8]. This is accompanied by the incidence of serious complications (e.g. stroke, acute renal failure, pulmonary complications, cancer and valve failure requiring hospitalization for heart failure) which significantly affect the quality of life [7]. According to a multivariate analysis of a cohort of 1038 patients listed in the European TAVI registry, the value of the logistic EuroSCORE, concomitant renal disease, liver disease and smoking were identified as variables with the highest hazard ratio for 1-year mortality [7]. Worsening of renal function and increased levels of N-terminal pro-B type natriuretic peptide (NT-proBNP) also helped predict an increased risk in TAVI patients [9], [10]. Standardized Valve Academic Research Consortium (VARC) endpoints definition for TAVI was used in our study as a useful and important tool for further research results' comparisons [11].

The aim of this pilot study was to investigate the added value of candidate biomarkers to standard clinical model in prediction of 30-day and 1-year risks of standardized endpoints, including mortality [11]. Another aim was to identify “non-responders” – i.e. patients in such a serious condition that even replacement of their stenotic valve would likely not affect their prognosis. This study analyzed biomarkers that have been previously published as prognostically significant in patients with cardiovascular disease. These substances can be divided into groups associated with 1) nitric oxide (NO): nitrite/nitrate and asymmetric dimethylarginine (ADMA); 2) oxidative stress: malondialdehyde (MDA), 8-hydroxy-2-deoxyguanosine (8-OHdG), the ferric-reducing ability of plasma (FRAP), cysteine, homocysteine, cysteinylglycine and glutathione; 3) the metabolism of extracellular matrix: matrix metalloproteinase 2 and 9 (MMP2, MMP9) and tissue inhibitors of metalloproteinases 1 (TIMP1); 4) renal functions: cystatin C; and 5) heart failure: B type natriuretic peptide (BNP) and NT-proBNP.

## Materials and Methods

The patients were followed according to protocol approved by the local ethics committee (Ethics Comittee of St. Ann University Hospital). All patients signed an informed consent before baseline investigations were performed and the study was conducted in accordance with the Helsinki declaration.

The prospective study comprised 45 patients with symptomatic severe aortic stenosis in whom SAVR was considered to be associated with a high risk of periprocedural death, as assessed by the logistic EuroSCORE. Baseline characteristics in the cohort of patients and their clinical outcomes have been published elsewhere [12]; therefore, methodology and clinical results are only briefly presented here. A complete set of biomarkers was acquired from 42 of the 45 patients included in the analysis. Since there was no difference in the hospitalization and long-term outcomes in patients treated with transfemoral (TF) vs. transapical (TA) approach, all patients treated with TAVI were evaluated together. Prior to operation, none of the patients were hemodynamically unstable, nor did they have resting angina, recent myocardial infarction (MI) or active endocarditis, and all procedures were performed electively. Contraindications included a bicuspid aortic valve, left ventricular ejection fraction ≤20% and severe (3+) mitral or aortic regurgitation. Additionally, patients with serious co-morbidities and an estimated life expectancy of less than three years were excluded from the study. Our multidisciplinary “Heart-team” (which consists of cardiac surgeons, interventional cardiologists and cardiologists specializing in echocardiography) assessed each patient and then decided upon a procedure to be performed in each individual. SAVR was not recommended for patients with serious clinical co-morbidities which were not measurable by the EuroSCORE (worsening mental status, limited physical activity, etc.). In the TAVI groups, the TA approach was the second choice in case the TF was impossible to perform. Depending on which procedure was used, patients were divided into SAVR (n = 13), TF (n = 14) and TA (n = 15) groups. Coronary artery disease did not influence the choice between SAVR and TAVI (seven patients had percutaneous coronary intervention and one patient had coronary artery bypass grafting before the procedure). TAVI procedures were performed using the technique described in medical literature [13], [14] and with the use of either Edwards-Sapien or Sapien XT aortic valves. All TAVI patients were given general anesthesia. Those in the TF group underwent surgical preparation of the common femoral artery. After the procedure, patients received dual anti-platelet therapy with acetylsalicylic acid (100 mg daily) and clopidogrel (75 mg daily). SAVR was performed during cardiac arrest that was induced by crystalloid cardioplegia. The bioprosthetic valve was sutured in a supra-annular position using pledgeted sutures situated on the ventricular side. Patients received post-operative warfarin therapy for 3 months. Both the TAVI and SAVR groups of patients were followed for 12 months. Each 3-month visit included a clinical examination, laboratory tests and transthoracic echocardiography. Monitored endpoints were defined based on the VARC (Valve Academic Research Consortium): 1) combined safety endpoint at 30 days; and 2) combined efficacy endpoint at 1 year (evaluation of device success was not included in this publication) [11]. A combined endpoint was defined as the occurrence of any clinical endpoint (either safety or efficacy endpoint) at 0–365 days. The combined endpoint analysis reflected the amount of patients experiencing at least one clinical endpoint. In the SAVR group of patients, the application of erythrocyte transfusion during extracorporeal circulation surgery was not considered to be a bleeding endpoint.

The biomarker blood samples were obtained from fasting subjects in the morning on the day of the operation. The samples were immediately cooled to 0°C, centrifuged in a refrigerated centrifuge and frozen at −70°C within 30 minutes after the draw. Values of creatinine and hemoglobin were evaluated promptly after the draw.

BNP was analyzed using the AxSYM BNP-Microparticle Enzyme Immunoassay (Abbott Laboratories, Abbott Park, USA). NT-ProBNP was analyzed using the Cobas E411 NT-proBNP Imunoassay Kit (Roche Diagnostics, Indianapolis). Cystatin C was analyzed using DakoCytomation Cystatin C Immunoparticles (DakoCytomation, Denmark). Plasma ADMA levels were measured in duplicate by commercially available ELISA kits (DLD Diagnostika Ltd, Germany) in accordance with the manufacturer's instructions. The range of minimum detection was 0.05 mmol/L, interassay coefficients of variation was <10%. Plasma 8-OHdG levels were analyzed using a Cayman Chemical EIA kit (Cayman Chemical Co., MI, USA) and following the manufacturer's protocol. The detection limit was 35 pg/L, and interassay CV% was <9%. To assess plasma antioxidant capacity, FRAP was measured according to the methods described by Benzie and Strain, only with one modification in procedure, i.e. absorbace was measured in a 96-well plates at 600 nm on Spectramax 340PC microplate reader (Molecular Devices, CA, USA). FRAP values were calculated by using ascorbic acid as the standard [15]. Plasma nitric oxide levels were evaluated by measuring the intermediate and end products - nitrite/nitrate (NOx) were measured using a colorimetric assay kit based on the Griess reaction (R&D Systems Inc., MN, USA). The assay sensitivity was 0.25 µmol/L, interassay precisions was <4.6%. The plasma level of MDA was assessed after derivation by thiobarbituric acid (TBA), using a modified high performance liquid chromatography with fluorescence detection as described previously [16]. Briefly, plasma aliquots were mixed with solutions of phosphoric acid (0.66 mol/L) and TBA (0.21 mol/L) and ultrapure water at a volume ratio of 1∶10∶10∶10. The mixture was heated at 95°C for 60 min. After cooling on ice, the mixture was neutralized using equal volumes of alkaline methanol and then centrifuged. The supernatant was injected onto a LiChrosorb RP18 (150 mm×4.6 mm, i.d.) column (Merck KGaA, Germany) with a mobile phase containing 50 mmol/L phosphate buffer, pH 6.8 and methanol (40∶60, v/v). Separated MDA-TBA adducts were quantified fluorometrically with excitation at 527 nm and emission at 551 nm. The limit of detection (signal/noise 5∶1) was 0.01 µmol/L. The interassay coefficients of variation was <4.2%.

The plasma totals of cysteine, homocysteine, cysteinylglycine and total glutathione concentrations were measured using modified high performance liquid chromatography with fluorescence detection (Shimadzu 10A series HPLC system, Shimadzu Corp., Japan) and the protocol described by Vester and Rasmussen. Briefly, the method applied tributylphosphine for reduction of thiols and ammonium 7-fluorobenzo-2-oxa-1,3-diazole-4-sulfonate as the derivatization agent. The limit of detection (signal/noise 5∶1) of homocysteine and glutathione was 0.2 µmol/L and 0.1 µmol/L, respectively. The interassay coefficients of variation was <3.2% [17]. Plasma MMP9 levels were determined in duplicate using a commercially available ELISA kit (GE Healthcare Life Sciences, USA). Serum TIMP1 and MMP2 levels were determined in duplicate using a commercially available ELISA kit (GE Healthcare Life Sciences, USA). The range of inter-assay CV% was <10%. The minimal detection sensitivity of the TIMP1 ELISA kit was 1.25 ng/mL, that of the MMP9 ELISA kit was 0.6 ng/mL, and the MMP2 ELISA kit was 0.37 ng/mL.

### Statistical Analysis

Standard descriptive statistics were used for analysis; continuous parameters were described by median and 5^th^–95^th^ percentiles, and categorical parameters were described by their absolute and relative frequencies. Differences between SAVR and TAVI groups were assessed using the Mann-Whitney test when parameters were continuous, and ML Chi square test was used for categorical parameters.

Receiver operating characteristic (ROC) curves and its area under curve (AUC) were used to determine if parameters were a good predictor for selected endpoints. We also used the net reclassification improvement (NRI) and the integrated discrimination improvement (IDI) to assess additional benefit of biomarkers to currently used clinical model (EuroSCORE) for prediction of the combined endpoint. The results of IDI were preferred in interpretation due their independence on cut-offs of calculated risk and usage of quantitative changes of calculated risk in their computation. The results of comparison of reference and improved models were visualized using risk assessment plots [18]. The level of statistical significance was set at α = 0.05. IBM SPSS 19 for Windows (Release 19.0.1, IBM Corporation 2010) was used for data analysis.

## Results

Baseline characteristics of patients are listed in Table 1. Characteristics of patients treated by transfemoral and transapical approach were comparable [12], only patients treated by TA approach had more often symptomatic peripheral vascular disease (3 patients in TA group vs. 0 patient in TF group) and more often insignificant atherosclerosis or tortuosity of pelvic arteries. Values of all tested biomarkers in both TAVI groups were comparable and due to the limited size of groups all analyses were performed for the whole TAVI group regardless of the approach. We calculated the logistic EuroSCORE II that was significantly lower than the original EuroSCORE. Laboratory values obtained prior to the procedure are shown in Table 2. The values of biomarkers observed in both groups did not differ from each other, only values of natriuretic peptides were slightly higher in patients treated by TAVI. The incidence of standardized endpoints and their combinations were comparable between the groups of patients treated by TAVI and SAVR (Table 3).

**Table 1:** Baseline characteristics.

	Total^1^	SAVR^1^	TAVI^1^	
	N = 42	N = 13	N = 29	Sig.^2^
**EuroSCORE**	21 (12; 35)	18 (12; 28)	24 (12; 37)	**0.025** *
**EuroSCORE II (2011)**	4.21 (1.84; 9.34)	3.81 (1.09; 7.35)	4.26 (1.85; 10.20)	0.295
**Age (years)**	82 (75; 89)	83 (76; 89)	82 (70; 89)	0.870
**Gender (male)**	13 (31.0%)	5 (38.5%)	8 (27.6%)	0.485
**Creatinine clearance** 3 **(mL/min)**	49.9 (29.7; 85.3)	56.8 (34.6; 85.3)	45.8 (26.9; 87.1)	0.153
**Extracardiac arteriopathy**	10 (23.8%)	4 (30.8%)	6 (20.7%)	NS
**Poor mobility**	3 (7.1%)	1 (7.7%)	2 (6.9%)	NS
**Previous cardiac surgery**	1 (2.4%)	0 (0.0%)	1 (3.4%)	NS
**Chronic lung disease**	6 (14.3%)	1 (7.7%)	5 (17.2%)	NS
**Diabetes mellitus on insulin**	4 (9.5%)	1 (7.7%)	3 (10.3%)	NS
**BMI (kg/m^2^)**	27.6 (20.8; 34.4)	28.7 (20.6; 38.9)	27.4 (20.8; 33.8)	0.248
**History of TIA/stroke**	7 (16.7%)	3 (23.1%)	4 (13.8%)	NS
**Smoking**	4 (9.5%)	0 (0.0%)	4 (13.8%)	0.076
**NYHA II**	6 (14.3%)	3 (23.1%)	3 (10.3%)	0.102
**NYHA III**	26 (61.9%)	8 (61.5%)	18 (62.1%)	
**NYHA IV**	10 (23.8%)	2 (15.4%)	8 (27.6%)	
**EF LV (%)**	57 (35; 75)	59 (51; 80)	57 (35; 72)	0.383
**PA systolic 0–30 mmHg**	14 (33.3%)	7 (53.8%)	7 (24.1%)	0.055
**PA systolic 31–55 mmHg**	16 (38.1%)	5 (38.5%)	11 (37.9%)	
**PA systolic >55 mmHg**	12 (28.6%)	1 (7.7%)	11 (37.9%)	
**Echocardiography**				
**Peak gradient (mmHg)**	83 (57; 131)	96 (57; 132)	80 (54; 117)	0.264
**Mean gradient (mmHg)**	50 (33; 80)	57 (35; 80)	50 (32; 71)	0.119
**AVA (cm^2^)**	0.60 (0.40; 0.96)	0.60 (0.45; 0.97)	0.57 (0.39; 0.96)	0.270
**AVA index**	0.34 (0.20; 0.50)	0.40 (0.20; 0.52)	0.30 (0.20; 0.45)	0.070
**Ejection fraction (%)**	57 (35; 75)	59 (51; 80)	57 (35; 72)	0.383
**LV diastolic diameter (mm)**	46 (39; 54)	46 (39; 55)	47 (37; 54)	0.785
**LV systolic diameter (mm)**	32 (23; 42)	32 (23; 39)	32 (23; 45)	0.754

1 Categorical parameters are described by absolute number and percentage of patients in given category; continuous variables are described by median (5^th^; 95^th^ percentile).

2 Overall statistical significance of differences among groups is based on Mann-Whitney test for continuous variables and ML chi-square test for categorical variables,

3 Creatinine clearance was estimated according to MDRD formula;

* statistically significant.

BMI – Body mass index, TIA – Transitory ischemic attack, EF LV – Ejection fraction of left ventricle, PA systolic – Pulmonary artery systolic pressure, AVA – Aortic valve area.

**Table 2:** Laboratory characteristics of SAVR and TAVI groups.

	Total^1^	SAVR^1^	TAVI^1^	
	N = 42	N = 13	N = 29	Sig.^2^
**Hemoglobin (g/L)**	126 (104; 150)	129 (105; 160)	126 (99; 149)	0.663
**BNP (pg/mL)**	328 (95; 1 868)	249 (34; 661)	388 (138; 2 212)	0.006*
**NT-proBNP (pg/mL)**	2 500 (263; 14 449)	1 308 (121; 12 501)	2 830 (371; 16 205)	0.007*
**Creatinine (µmol/L)**	90 (57; 149)	91 (57; 138)	89 (57; 165)	0.734
**Cystatin C (mg/ml)**	1.68 (1.15; 2.69)	1.73 (1.15; 2.49)	1.62 (1.11; 3.08)	0.785
**ADMA (µmol/L)**	0.76 (0.56; 0.95)	0.74 (0.64; 0.92)	0.77 (0.41; 1.83)	0.870
**Nitrite/nitrate (µmol/L)**	53 (20; 100)	54 (18; 211)	52 (20; 100)	0.860
**8-OHdG (µg/L)**	10.5 (6.3; 17.8)	9.2 (5.8; 15.1)	10.9 (6.3; 19.9)	0.314
**FRAP (µmol/L)**	1 008 (656; 1 712)	935 (688; 1 266)	1 029 (623; 1 851)	0.215
**MDA (µmol/L)**	0.51 (0.32; 0.93)	0.42 (0.33; 0.81)	0.54 (0.31; 0.93)	0.757
**Cysteine (µmol/L)**	409 (311; 547)	423 (212; 526)	401 (311; 566)	0.875
**Homocysteine (µmol/L)**	18.6 (9.4; 37.5)	17.2 (6.6; 33.1)	18.6 (9.4; 39.3)	0.667
**Cysteinyl-Glycine (µmol/L)**	41.9 (26.8; 61.5)	43.0 (26.8; 61.8)	41.9 (25.0; 59.3)	0.808
**Glutathione (µmol/L)**	1.94 (1.09; 4.05)	2.11 (1.09; 8.07)	1.80 (0.98; 3.58)	0.218
**MMP-2 (ng/mL)**	143 (98; 263)	134 (75; 197)	143 (98; 268)	0.374
**MMP-9 (ng/mL)**	1 538 (536; 1 878)	1 514.5 (468; 1 831)	1 538 (537; 1 880)	0.883
**TIMP-1 (ng/mL)**	0.57 (0.15; 1.34)	0.35 (0.14; 1.18)	0.65 (0.16; 1.43)	0.166

1 Parameters are described by median (5^th^; 95^th^ percentile).

2 Overall statistical significance of differences among groups is based on Mann-Whitney test for continuous variables;

* statistically significant.

BNP – B type natriuretic peptide, NT-proBNP – N-terminal pro-B type natriuretic peptide, ADMA – asymmetric dimethylarginine.

**Table 3:** Occurrence of endpoints in SAVR and TAVI groups.

	Total^1^	SAVR^1^	TAVI^1^	
	N = 42	N = 13	N = 29	Sig.^2^
**Combined safety endpoint (at 30 days)**	8 (19.0%)	3 (23.1%)	5 (17.2%)	0.660
Major stroke	2 (4.8%)	0 (0.0%)	2 (6.9%)	0.217
Life-threatening bleeding	1 (2.4%)	0 (0.0%)	1 (3.4%)	0.386
All-cause mortality	1 (2.4%)	1 (7.7%)	0 (0.0%)	0.121
Acute kidney injury (stage 3)	2 (4.8%)	0 (0.0%)	2 (6.9%)	0.217
Peri-procedural MI	4 (9.5%)	2 (15.4%)	2 (6.9%)	0.403
Major vascular complication	1 (2.4%)	0 (0.0%)	1 (3.4%)	0.386
Repeat procedure for valve-related dysfunction	1 (2.4%)	0 (0.0%)	1 (3.4%)	0.386
**All-cause mortality at 1 year**	6 (14.3%)	2 (15.4%)	4 (13.8%)	0.892
**Combined endpoint (0–365 days)**	15 (35.7%)	6 (46.2%)	9 (31.0%)	0.349

*Without one patient (deceased within 30 days)*	N = 41	N = 12	N = 29	
**Combined efficacy endpoint (31–365 days)**	8 (19.5%)	3 (25.0%)	5 (17.2%)	0.575
All-cause mortality -30–365 days	5 (12.2%)	1 (8.3%)	4 (13.8%)	0.616
Failure of current therapy for AS, requiring hospitalization due to symptoms of valve-related or cardiac decompensation	2 (4.9%)	0 (0.0%)	2 (6.9%)	0.232
Prosthetic heart valve dysfunction (aortic valve area <1.2 cm2 and mean aortic gradient ≥20 mmHg or peak velocity ≥3 m/s or moderate or severe prosthetic valve AR)	2 (6.3%)	2 (20.0%)	0 (0.0%)	0.026*

1 Categorical parameters are described by absolute number and percentage of patients in given category.

2 Overall statistical significance of differences among groups is based on ML chi-square test;

* statistically significant.

The receiver operating characteristic analysis (ROC) was performed for all biomarkers for the TAVI group (Table 4) and for all patients (Table 5). Biomarkers with significant results (p≤0.05) are presented in Table 6 together with the values of area under the curve (AUC), sensitivity, specificity and cut-off values for TAVI group and for the whole population (TAVI group and SAVR group).

**Table 4:** ROC analysis for prediction of endpoints in patients treated with TAVI.

	1-year mortality		Combined safety endpoint at 30 days		Combined efficacy endpoint 31–365 days		Combined endpoint 0–356 days	
	N = 4 (13.8%)		N = 5 (17.2%)		N = 5 (17.2%)		N = 9 (31.0%)	
	Sig.	AUC (95% CI)	Sig.	AUC (95% CI)	Sig.	AUC (95% CI)	Sig.	AUC (95% CI)
**EuroSCORE**	0.015*	**0.885 (0.714; 1.000)**	0.119	0.725 (0.518; 0.932)	0.126	0.721 (0.405; 1.000)	0.063	0.719 (0.510; 0.929)
**EuroSCORE II**	0.359	0.645 (0.396; 0.894)	0.112	0.729 (0.489; 0.969)	0.237	0.671 (0.454; 0.887)	0.053	0.728 (0.537; 0.919)
**BNP**	0.100	0.760(0.563;0.957)	0.817	0.467 (0.271; 0.663)	0.065	0.767(0.589;0.945)	0.172	0.661(0.467;0.855)
**NTproBNP**	0.184	0.710(0.534;0.886)	1.000	0.500 (0.274; 0.726)	0.073	0.758(0.591;0.925)	0.172	0.661(0.455;0.868)
**Creatinine clearance**	-	-	0.273	0.658 (0.468; 0.849)	0.729	0.550 (0.276; 0.824)	0.300	0.622 (0.411; 0.833)
**Cystatin C**	1.000	0.500 (0.240; 0.760)	0.931	0.513 (0.249; 0.776)	0.862	0.475 (0.246; 0.704)	0.795	0.469 (0.259; 0.679)
**ADMA**	0.242	0.685 (0.477; 0.893)	0.436	0.388 (0.044; 0.731)	0.341	0.638 (0.429; 0.846)	0.671	0.550 (0.321; 0.779)
**Nitrite/Nitrate**	0.613	0.420 (0.169; 0.671)	0.773	0.458 (0.218; 0.699)	0.686	0.442 (0.211; 0.672)	0.777	0.533 (0.322; 0.744)
**8-OHdG**	0.343	0.650 (0.362; 0.938)	0.403	0.621 (0.396; 0.845)	0.285	0.654 (0.410; 0.898)	0.289	0.625 (0.422; 0.828)
**FRAP**	0.555	0.594 (0.281; 0.907)	0.088	0.771 (0.599; 0.942)	0.368	0.630 (0.365; 0.896)	0.121	0.691 (0.485; 0.896)
**MDA**	0.149	0.729 (0.493; 0.965)	0.004*	**0.958 (0.878; 1.000)**	0.093	0.743 (0.540; 0.947)	0.002*	**0.872 (0.727; 1.000)**
**Cysteine**	0.327	0.655 (0.355; 0.955)	0.030*	**0.813 (0.621; 1.000)**	0.885	0.479 (0.151; 0.807)	0.203	0.650 (0.417; 0.883)
**Homocysteine**	0.924	0.515 (0.224; 0.806)	0.356	0.633 (0.374; 0.893)	0.840	0.471 (0.212; 0.729)	0.981	0.503 (0.287; 0.718)
**Cysteinylglycine**	0.569	0.590 (0.353; 0.827)	0.817	0.533 (0.215; 0.851)	0.273	0.658 (0.437; 0.880)	0.346	0.611 (0.379; 0.843)
**Glutathion**	0.327	0.345 (0.033; 0.657)	0.729	0.550 (0.258; 0.842)	0.194	0.688 (0.422; 0.953)	0.588	0.564 (0.319; 0.808)
**TIMP1**	0.376	0.640 (0.378; 0.902)	0.013*	**0.858 (0.723; 0.994)**	0.908	0.483 (0.190; 0.776)	0.053	0.728 (0.511; 0.944)
**MMP2**	0.511	0.396 (0.190; 0.601)	0.005*	**0.948 (0.858; 1.000)**	0.610	0.426 (0.223; 0.629)	0.022*	**0.781 (0.605; 0.957)**
MMP9	0.167	0.818 (0.576; 1.000)	0.079	0.800 (0.541; 1.000)	0.398	0.667 (0.342; 0.992)	0.086	0.786 (0.523; 1.000)

**Table 5:** ROC analysis for prediction of endpoints in patients treated with SAVR and TAVI.

	1-year mortality		Combined safety endpoint at 30 days		Combined efficacy endpoint 31–365 days		Combined endpoint 0–356 days	
	N = 6 (14.3%)		N = 8 (19.0%)		N = 8 (19.5%)		N = 15 (35.7%)	
	Sig.	AUC (95% CI)	Sig.	AUC (95% CI)	Sig.	AUC (95% CI)	Sig.	AUC (95% CI)
**EuroSCORE**	0.014*	**0.817 (0.654; 0.980)**	0.120	0.678 (0.491; 0.866)	0.490	0.580 (0.321; 0.838)	0.203	0.620 (0.440; 0.800)
**EuroSCORE II**	0.289	0.637 (0.423; 0.850)	0.066	0.711 (0.507; 0.916)	0.162	0.661 (0.485; 0.837)	0.027*	**0.709 (0.550; 0.867)**
**BNP**	0.376	0.614(0.343;0.885)	0.844	0.523(0.332;0.714)	0.437	0.590(0.353;0.827)	0.432	0.574(0.394;0.755)
**NTproBNP**	0.590	0.569(0.327;0.812)	0.848	0.522(0.327;0.717)	0.469	0.583(0.366;0.800)	0.520	0.560(0.380;0.741)
**Creatinine clearance**	0.719	0.546 (0.332; 0.761)	0.063	0.713 (0.562; 0.864)	0.717	0.542 (0.326; 0.757)	0.131	0.642 (0.472; 0.812)
**Cystatin C**	0.685	0.552 (0.350; 0.755)	0.300	0.619 (0.417; 0.821)	0.839	0.477 (0.289; 0.664)	0.617	0.547 (0.373; 0.722)
**ADMA**	0.398	0.609 (0.418; 0.799)	0.923	0.489 (0.227; 0.751)	0.490	0.580 (0.402; 0.758)	0.969	0.496 (0.315; 0.678)
**Nitrite/nitrate**	0.408	0.394 (0.157; 0.630)	0.195	0.649 (0.441; 0.857)	0.755	0.536 (0.337; 0.735)	0.394	0.580 (0.404; 0.757)
**8-OHdG**	0.258	0.646 (0.397; 0.894)	0.019*	**0.770 (0.618; 0.923)**	0.908	0.513 (0.289; 0.737)	0.128	0.643 (0.472; 0.814)
**FRAP**	0.337	0.634 (0.381; 0.887)	0.017*	**0.809 (0.677; 0.940)**	0.243	0.635 (0.427; 0.843)	0.019*	**0.731 (0.568; 0.894)**
**MDA**	0.198	0.680 (0.462; 0.898)	0.013*	**0.821 (0.593; 1.000)**	0.120	0.680 (0.500; 0.859)	0.007*	**0.765 (0.608; 0.922)**
**Cysteine**	0.135	0.708 (0.455; 0.962)	0.007*	**0.809 (0.655; 0.963)**	0.887	0.483 (0.231; 0.734)	0.074	0.672 (0.494; 0.850)
**Homocysteine**	0.392	0.619 (0.361; 0.878)	0.025*	**0.758 (0.578; 0.938)**	0.915	0.487 (0.282; 0.692)	0.221	0.618 (0.445; 0.790)
**Cysteinylglycine**	0.232	0.667 (0.471; 0.862)	0.130	0.674 (0.439; 0.910)	0.606	0.563 (0.362; 0.764)	0.161	0.635 (0.452; 0.818)
**Glutathione**	0.765	0.458 (0.124; 0.792)	0.308	0.617 (0.394; 0.841)	0.062	0.727 (0.525; 0.929)	0.660	0.542 (0.346; 0.738)
**TIMP1**	0.461	0.595 (0.368; 0.822)	0.003*	**0.845 (0.724; 0.965)**	0.685	0.547 (0.297; 0.796)	0.058	0.679 (0.492; 0.867)
**MMP2**	0.940	0.510 (0.311; 0.709)	0.219	0.649 (0.382; 0.916)	0.972	0.496 (0.288; 0.704)	0.411	0.580 (0.387; 0.773)
**MMP9**	0.101	0.861 (0.678; 1.000)	0.117	0.726 (0.477; 0.976)	0.637	0.578 (0.244; 0.912)	0.239	0.657 (0.411; 0.902)

**Table 6:** Receiver operating characteristic (ROC) analysis for selected biomarkers.

TAVI (N = 29)	AUC (95% IS)	Sig.	Sensitivity	Specificity	Cut-off
***1-year mortality***					
EuroSCORE	0.885 (0.714; 1.000)	0.015*	0.750	0.960	≥33.5
***Combined safety endpoint at 30 days***					
MDA	0.958 (0.878; 1.000)	0.004*	1.000	0.958	≥0.795
TIMP1	0.858 (0.723; 0.994)	0.013*	1.000	0.750	≥160
MMP2	0.948 (0.858; 1.000)	0.005*	1.000	0.833	≥1693
Cysteine	0.813 (0.621; 1.000)	0.030*	0.600	0.958	≥517
***Combined efficacy endpoint 31–365 days***					
BNP	0.767 (0.589; 0.945)	0.065	0.800	0.792	≥744
NTproBNP	0.758 (0.591; 0,925)	0.073	1.00	0.667	≥3452
***Combined endpoint 0–365 day*** *s*					
MDA	0.872 (0.727; 1.000)	0.002*	0.875	0.850	≥0.585
MMP2	0.781 (0.605; 0.957)	0.022*	1.000	0.550	≥1215

*SAVR+TAVI (N = 42)*	AUC (95% IS)	Sig.	Sensitivity	Specificity	Cut-off
***1-year mortality***					
EuroSCORE	0.817 (0.654; 0.980)	0.014*	0.833	0.694	≥24.5
***Combined safety endpoint at 30 days***					
8-OHdG	0.770 (0.618; 0.923)	0.019*	0.625	0.882	≥14.25
FRAP	0.809 (0.677; 0.940)	0.017*	1.000	0.676	≥1055
MDA	0.821 (0.593; 1.000)	0.013*	0.833	0.824	≥0.625
Cysteine	0.809 (0.655; 0.963)	0.007*	1.000	0.515	≥400
Homocysteine	0.758 (0.578; 0.938)	0.025*	0.875	0.636	≥19.0
TIMP1	0.845 (0.724; 0.965)	0.003*	0.875	0.788	≥158.7
***Combined endpoint (0–365 days)***					
EuroSCORE II	0.709(0.550;0.867)	0.027*	0.667	0.741	≥4.675
FRAP	0.731 (0.568; 0.894)	0.019*	0.769	0.741	≥1066
MDA	0.765 (0.608; 0.922)	0.007*	0.692	0.815	≥0.585

In the group of patients treated using TAVI we identified a higher risk of the combined safety endpoint at 30 days for increased values of markers of oxidative stress - MDA and cysteine, and for the increased values of markers associated with extracellular matrix metabolism - MMP2 and TIMP1. Similar results were obtained for the whole study population (TAVI group and SAVR group). Moreover, also higher levels of 8-OHdG and homocysteine but not MMP2 turned out to be significant markers of the combined safety endpoint at 30 days. Increased levels of MDA and MMP2 turned out to be significant in prediction of all endpoints at 1 year in the TAVI group and higher levels of MDA and FRAP were found to be associated with prediction of all endpoints at 1 year for all patients. According to ROC analysis there was a remarkable but statistically insignificant association between increased levels of both natriuretic peptides and higher incidence of efficacy endpoints during 30–365 days of follow-up in the TAVI group.

Higher EuroSCORE predicted higher 1-year mortality for the TAVI group and also for all patients. Higher EuroSCORE II was associated with prediction of endpoints at 1 year for all patients.

**Figure 1:**
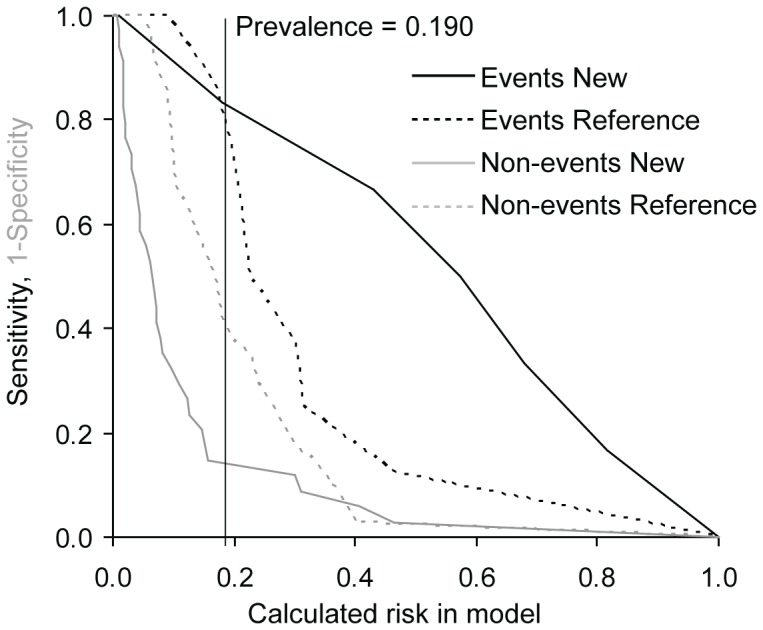
Risk assessment plot of the performance comparison between reference EuroSCORE model and new EuroSCORE+MDA model for combined safety endpoint at 30 days in TAVI+SAVR group (N = 42). Prevalence – occurrence of endpoint in given group.

**Figure 2:**
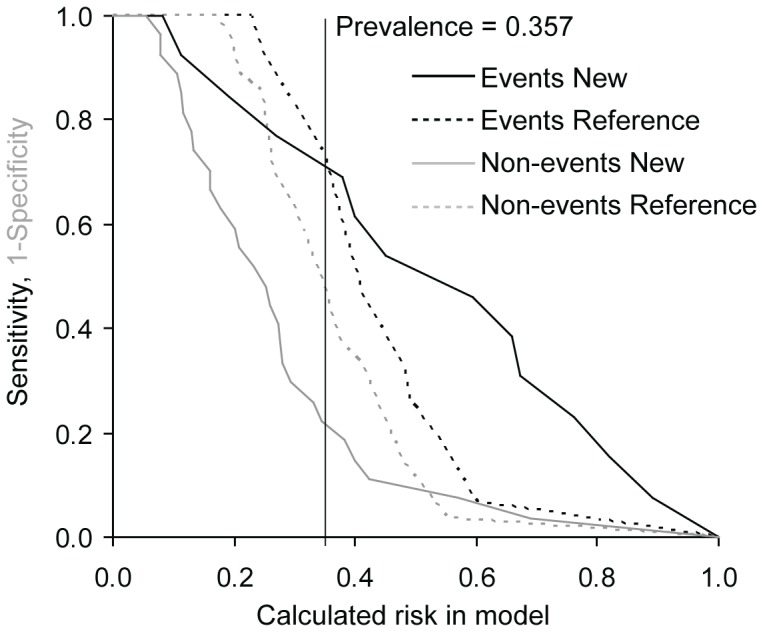
Risk assessment plot of the performance comparison between reference EuroSCORE model and new EuroSCORE+MDA model for combined endpoint at 30 days in TAVI+SAVR group (N = 42).

**Figure 3:**
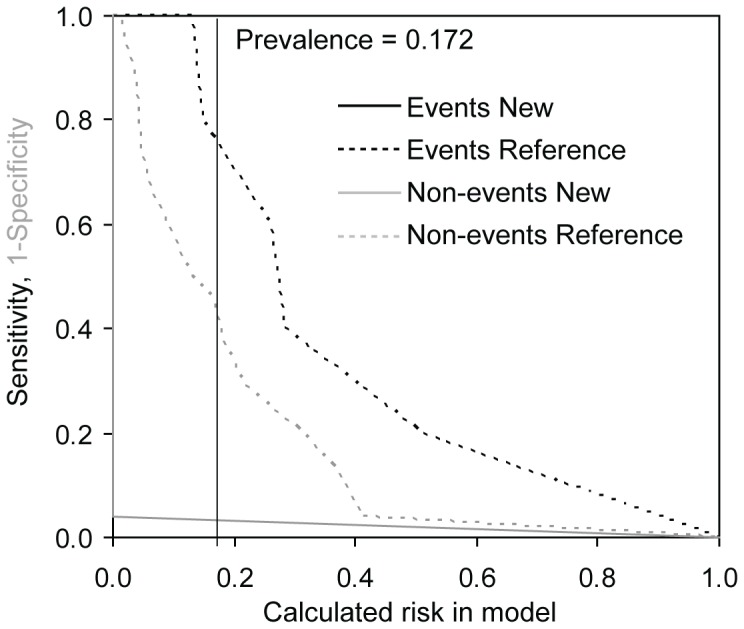
Risk assessment plot of the performance comparison between reference EuroSCORE model and new EuroSCORE+MDA model for combined safety endpoint at 30 days in TAVI group (N = 29).

**Figure 4:**
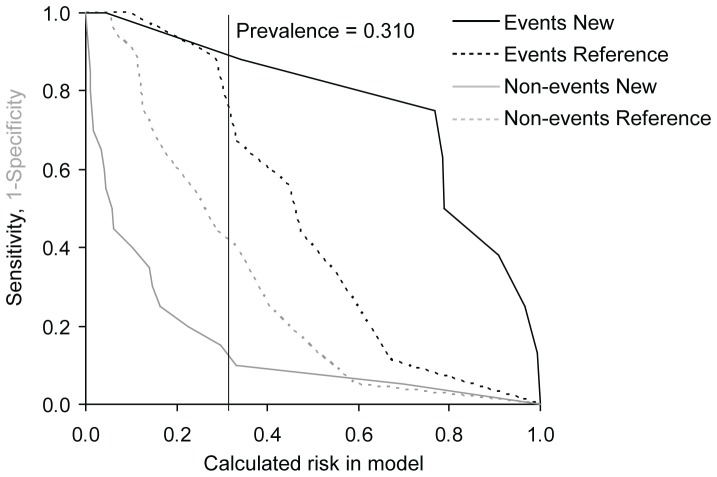
Risk assessment plot of the performance comparison between reference EuroSCORE model and new EuroSCORE+MDA model for combined endpoint at 30 days in TAVI group (N = 29).

To determine whether the consideration of values of candidate biomarkers in addition to currently used clinical model improves our possibilities of risk prediction we used NRI and IDI. As the gold clinical standard, the EuroSCORE was used and only biomarkers with significant predictive value according to ROC analysis were evaluated. In the TAVI group we found improved reclassification and discrimination of the clinical model for MDA and MMP2 for prediction of the combined safety endpoint at 30 days and the combined endpoint (0–365 days) and improved reclassification for TIMP1 for prediction of the combined safety endpoint at 30 days. In the group of SAVR and TAVI we detected improved reclassification for 8-OHdG, MDA and TIMP1 for prediction of the combined safety endpoint at 30 days and for FRAP for the combined endpoint (0–365 days) and improved discrimination for MDA for both the combined safety endpoint at 30 days and the combined endpoint (0–365 days) (Table 7). The results for MDA models as the most important addition to reference EuroSCORE model are visualized in risk assessment plots of the performance comparisons between reference and new models (Figures 1, 2, 3, and 4).

**Table 7:** Evaluation of additional benefit of biomarkers to clinical model (EuroSCORE) by net reclassification improvement (NRI) and the integrated discrimination improvement (IDI) for prediction of combined safety endpoint at 30 days and combined endpoint (0–365 days) in groups of patients treated by TAVI and by SAVR and TAVI.

TAVI (N = 29)	IDI (95% CI)	p	NRI (95% CI)	P
***Combined safety endpoint at 30 days***				
MDA	0.891 (0.710; 1.071)	**<0.001**	1.292 (0.393; 2.190)	**0.005**
TIMP1	0.071 (−0.031; 0.173)	0.170	0.608 (0.025; 1.192)	**0.041**
MMP2	0.852 (0.694; 1.011)	**<0.001**	1.292 (0.393; 2.190)	**0.005**
Cysteine	0.043 (−0.030; 0.117)	0.248	0.367 (−0.073; 0.807)	0.102
***Combined endpoint 0–365 day*** *s*				
MDA	0.439 (0.217; 0.662)	**<0.001**	0.825 (0.363; 1.287)	**<0.001**
MMP2	0.177 (0.056; 0.298)	**0.004**	0.400 (0.090; 0.710)	**0.011**

*SAVR+TAVI (N = 42)*	IDI (95% CI)	p	NRI (95% CI)	P
***Combined safety endpoint at 30 days***				
8-OHdG	0.062 (−0.016; 0.141)	0.120	0.456 (0.036; 0.876)	**0.033**
FRAP	0.017 (−0.045; 0.078)	0.595	0.284 (−0.099; 0.667)	0.146
MDA	0.290 (0.125; 0.455)	**<0.001**	0.853 (0.226; 1.480)	**0.008**
Cysteine	0.079 (−0.005; 0.163)	0.064	0.500 (−0.045; 1.045)	0.072
Homocysteine	0.067 (−0.027; 0.161)	0.165	0.466 (−0.024; 0.956)	0.062
TIMP1	0.087 (−0.007; 0.181)	0.071	0.591 (0.050; 1.132)	**0.032**
***Combined endpoint (0–365 days)***				
FRAP	0.030 (−0.040; 0.100)	0.401	0.416 (0.092; 0.740)	**0.012**
MDA	0.188 (0.059; 0.316)	**0.004**	0.256 (−0.127; 0.640)	0.190

## Discussion

This work is the first to evaluate the possibility of using biomarkers other than creatinine and natriuretic peptides in prediction of prognosis of high-risk patients treated for significant aortic stenosis. The knowledge of values of several biomarkers may bring another piece of information into the decision-making process and, in addition to clinical parameters; helps select the patients in the highest risk of subsequent adverse events.

### Clinical Scoring Systems

Due to low in-hospital mortality, we did not evaluate the predictive values of logistic EuroSCOREs I and II in terms of short-term mortality in TAVI patients. Another study with a more extensive group of TAVI patients showed that neither the logistic EuroSCORE [19] nor the STS-PROM score [20] precisely predicted periprocedural complications or mortality. There are still no publications describing the predictive value of the logistic EuroSCORE II for in-hospital mortality with TAVI procedures. According to our results, both logistic EuroSCORE and EuroSCORE II could be used as scoring systems for prediction of annual mortality or incidence of standardized combined endpoints following TAVI but with limited sensitivity and specificity. So far, papers using standardized endpoints defined according to VARC [11] that will enable a comparison of individual studies in the future have been published only sporadically [21].

In comparison with the previous study [7], we found only a trend for impaired renal function to be a predictor of the combined safety endpoint at 30 days with a cut-off value for creatinine clearance of 46 ml/min (Table 4b). We assume that a larger study group would have led to significant results. In our study we did not evaluate smoking status (due to low number of smokers – only 4 patients) or liver disease as possible predictors of adverse prognosis. A multivariate model including clinical data was not computed because a model with more than 2 variables became unstable due to low sample size.

### Biomarkers

Previous studies have only evaluated the prognostic effect of natriuretic peptides. Kefer et al. found that only BNP levels were independent predictors of 30-day survival [22] and Spargias et al. found that increased levels of NT-proBNP predicted higher 30-day and 1-year mortality. We found only an insignificant association between higher natriuretic peptides and occurrence of efficacy endpoints during 30–365 days. Lower number of treated patients and reduced incidence of endpoints in our study could have been the cause. In accordance with previous results it seems that high levels of natriuretic peptides are associated with adverse prognosis in high risk patients with severe aortic stenosis treated by TAVI. The main contribution of our work was the analysis of novel biomarkers and evaluation of their additional benefit to the standard clinical model (EuroSCORE) in prediction of post-operative adverse events.

### Biomarker Reflecting Renal Function

Cystatin C is mainly used as a biomarker of kidney function and describes glomerular filtration better than serum creatinine. In contrast to higher serum creatinine being a strong predictor of 30-day and 1-year mortality following TAVI [9], we did not find any similar association with cystatin C. Probably the post-operative values of cystatin C and its dynamics during the peri-operative course should be studied for early diagnostics of AKI or adverse prognosis after TAVI.

### Biomarkers Reflecting NO Production

ADMA is closely related to L-arginine and is a natural endogenous competitive inhibitor of NO synthase; nitrites/nitrates (NO2−/NO3−) are stable detectable products of NO. While low production of NO results in endothelial dysfunction and vasodilatation failure, high levels of NO, produced by inducible NO synthase (iNOS) during septic or cardiogenic shock, further deteriorate cardiac function and lead to severe vasodilatation and hypotension. In our work, we did not find any association between either pre-operative ADMA or nitrite/nitrate values, and the incidence of short and long-term endpoints in patients after SAVR or TAVI.

### Biomarkers Reflecting Oxidative Stress

Oxidative stress represents an imbalance between the production of reactive oxygen species (ROS) and the ability to detoxify reactive products or repair the resulting damage. Toxic effects of oxidative stress, caused through the production of peroxides and free radicals, lead to damage of all cell components, including proteins, lipids and DNA. Malondialdehyde (MDA) is a highly reactive compound and a marker of oxidative stress levels. According to our results, increased pre-operative values of MDA turned out to be a very promising biomarker of short and long-term outcomes in both the group of high-risk patients treated by TAVI, as well as the entire study population treated with either SAVR or TAVI. Even after adding to EuroSCORE, representing the standard clinical model, MDA improved reclassification and discrimination of the model; especially for calculated risk values above overall prevalence of endpoint. High levels of MDA might reflect severity of disease, loss of repair antioxidant function and, as a consequence, poor prognosis despite our aggressive and expensive treatment. 8-hydroxy-2-deoxyguanosine (8-OHdG) is a product of DNA oxidation and a biomarker of oxidative stress and DNA damage. Data has already been published demonstrating that in patients with heart failure the increased values correlate to the severity of heart failure, left ventricle dysfunction and natriuretic peptide values [23]. In our work, increased values of 8-OHdG that were found throughout the entire study population (both SAVR and TAVI groups) were associated with unfavorable 30-day prognosis following aortic valve replacement and improved discrimination of the clinical model. The ferric-reducing ability of plasma (FRAP) represents the total antioxidant capacity of plasma (a complex of all compounds with antioxidant effects, e.g. antioxidative vitamins, uric acid, bilirubin, proteins and other exogenous substances). According to our results, elevated pre-operative FRAP values were associated with a higher incidence of combined safety endpoints at 30 days. Thiols (cysteine, homocysteine, cysteinylglycine, glutathione, gamma-glutamyl-cysteinyl-glycine) are organosulfur compounds that contain reactive carbon-bonded sulfhydryl groups (-SH) that have antioxidant potential. We identified both elevated levels of cysteine and homocysteine as possible predictors of a higher incidence of combined safety endpoints at 30 days but when added to the clinical model the prognostic value did not improve.

### Biomarkers Reflecting the Metabolism of Extracellular Matrix (ECM)

Matrix metalloproteinases (MMPs) are enzymes involved in the metabolism of ECM. The tissue inhibitor of metalloproteinase 1 is one endogenous inhibitor of matrix metalloproteinases. It has been demonstrated that, in elderly men, serum MMP9 and TIMP1 levels were related to increased mortality risk [24], [25]. In our cohort, levels of MMP2 and TIMP1 were related to the incidence of combined safety endpoints at 30 days even when added to the clinical model. Altered extracellular matrix metabolism may be involved in left ventricle remodeling, as well as other detrimental pathways; therefore, circulating MMP2 or TIMP1 may be relevant biomarkers.

In this work we pointed out several biomarkers that could help us stratify patients undergoing TAVI. Although there are some experimental works suggesting a possibility to influence oxidative stress pharmacologically [26] we assume that the major benefit of biomarkers is to identify patients in the highest risk of post-operative adverse events. This precise stratification could point out those with the need for more intensive peri-operative care or bring up an indication for conservative therapy.

Major study limitations included the monocentric character of our prospective study and a limited number of enrolled patients. This fact was taken into consideration during the interpretation and discussion of statistical results. Therefore, it was not possible to analyze the prognostic significance of the biomarkers for each endpoint separately, and we had to use combined endpoints instead. Although outcomes forming combined endpoints are disparate, they reflect adverse course after TAVI in a very complex way and in our article we followed standardized endpoint definitions according to a consensus report from the Valve Academic Research Consortium [11]. Population size and incidence of endpoints were consistent with other studies that have evaluated the prognostic influence of NT-proBNP in patients treated with TAVI. From a statistical point of view, our study had several limitations and, as such, it should be regarded as a novel hypothesis generating study, setting the first step in further exploration of this highly relevant topic. Other limitations included using VARC defined endpoints standardized for TAVI also for the SAVR group, such as definitions of either periprocedural myocardial infarction or prosthetic heart valve dysfunction.

### Conclusions

Despite highly sophisticated TAVI or SAVR treatments for patients with severe aortic stenosis, at least 15% of patients die within one year and another 20% experience a serious event. In this study we sought to identify high risk patients using biomarkers and we found malondialdehyde to be the most promising biomarker, although other applicable parameters included 8-OHdG, FRAP, MMP2 and TIMP1.

Our results are in concordance with other studies, which have shown the necessity to create and validate a new clinical scoring system dedicated to TAVI that will help predict both short- and long-term mortality. The potential contribution of the proposed biomarkers (MDA, FRAP, MMP2, TIMP1, BNP and NTproBNP) must now be assessed in large multicentre studies, optimally in addition to clinical parameters included in the new “TAVIscore”.
